# Linking Transcriptional Changes over Time in Stimulated Dendritic Cells to Identify Gene Networks Activated during the Innate Immune Response

**DOI:** 10.1371/journal.pcbi.1003323

**Published:** 2013-11-07

**Authors:** Ashwini Patil, Yutaro Kumagai, Kuo-ching Liang, Yutaka Suzuki, Kenta Nakai

**Affiliations:** 1 The Institute of Medical Science, The University of Tokyo, Tokyo, Japan; 2 WPI Immunology Frontier Research Center, Osaka University, Osaka, Japan; 3 Department of Medical Genome Sciences, The University of Tokyo, Tokyo, Japan; University of Virginia, United States of America

## Abstract

The innate immune response is primarily mediated by the Toll-like receptors functioning through the MyD88-dependent and TRIF-dependent pathways. Despite being widely studied, it is not yet completely understood and systems-level analyses have been lacking. In this study, we identified a high-probability network of genes activated during the innate immune response using a novel approach to analyze time-course gene expression profiles of activated immune cells in combination with a large gene regulatory and protein-protein interaction network. We classified the immune response into three consecutive time-dependent stages and identified the most probable paths between genes showing a significant change in expression at each stage. The resultant network contained several novel and known regulators of the innate immune response, many of which did not show any observable change in expression at the sampled time points. The response network shows the dominance of genes from specific functional classes during different stages of the immune response. It also suggests a role for the protein phosphatase 2a catalytic subunit α in the regulation of the immunoproteasome during the late phase of the response. In order to clarify the differences between the MyD88-dependent and TRIF-dependent pathways in the innate immune response, time-course gene expression profiles from MyD88-knockout and TRIF-knockout dendritic cells were analyzed. Their response networks suggest the dominance of the MyD88-dependent pathway in the innate immune response, and an association of the circadian regulators and immunoproteasomal degradation with the TRIF-dependent pathway. The response network presented here provides the most probable associations between genes expressed in the early and the late phases of the innate immune response, while taking into account the intermediate regulators. We propose that the method described here can also be used in the identification of time-dependent gene sub-networks in other biological systems.

## Author Summary

The innate immune response is the first level of protection in organisms against invading pathogens. It consists of a large number of proteins functioning in signaling cascades triggered by the binding of fragments from microbes to specific cellular receptors. Disruptions in these pathways can lead to numerous diseases. As such, the innate immune system has been the subject of a large number of studies. However, due to its complexity and the interplay of a large number of pathways, it is not yet completely understood. In this study, we measured transcriptional changes in activated immune cells and used this information in the context of a large network of protein-protein and protein-DNA interactions to identify a smaller network of response genes. We did this by identifying the most probable network paths connecting genes showing large changes in their expression patterns at successive stages of the response. Analysis of this activated gene network revealed the associations between various temporal regulators of the innate immune response. We also identified response networks for immune cells lacking important mediators, MyD88 and TRIF, to clarify the distinct functional modules affected by their associated pathways in the innate immune response.

## Introduction

The innate immune system is the primary host response to invading pathogens. The innate immune response is characterized by germline-encoded pattern-recognition receptors (PRRs) that detect and bind to specific microbial components, also known as pathogen-associated molecular patterns (PAMPs). Toll-like receptors (TLRs) are a family of PRRs that are conserved from worm to mammals and expressed on different types of immune cells, such as macrophages, dendritic cells (DCs) and B cells, as well as non-immune cells, such as fibroblasts and epithelial cells. 10 and 13 TLRs have been identified in human and mouse, respectively, each with distinct microbial ligands. The binding of these ligands to their specific receptors triggers downstream signaling cascades causing the expression of pro-inflammatory cytokines, ultimately leading to systemic inflammation. TLRs primarily function through two pathways – the MyD88-dependent pathway which leads to the expression of proinflammatory cytokines, and the TIR-domain–containing adaptor protein-inducing IFN-β (TRIF)-dependent pathway which produces the type I interferons (IFNs) [1], [2].

Though much is known about the pathways activated during the innate immune response, recent perturbation studies have identified previously unknown regulators and transcription factors, highlighting the complexity of the innate immune system and the incompleteness of our current knowledge [3]–[5]. While these studies provide important information about the genes affected on perturbation of a causal gene, they do not explain the cause of the observed expression changes. Additionally, these studies are inherently limited to genes which show changes in expression at the time of observation thus providing an incomplete representation of the activated pathways. The complexity of the innate immune system, the ease of monitoring transcriptional changes, and the availability of large amounts of regulatory and interaction information, all facilitate its analysis using computational methods. An initial computational study mapped all the known interactions associated with the immune response from literature [6]. This study provided a high confidence signaling network and identified the “bow-tie” structure of the immune response. However, it was limited in size and coverage. Li et al. used this signaling map to identify 10 distinct input-output pathways [7]. The resultant modules were further used by Richard et al. to identify a minimum set of genes whose deletion affects the fidelity of the TLR signaling pathways [8]. Though these methods used novel approaches to analyze the TLR signaling pathways, they did not take the temporal changes of the immune response into account. Using a different approach, Seok et al. studied the regulatory networks of 10 transcription factors and their targets using the Network Component Analysis approach [9]. While this study considered the dynamic nature of the immune response through the use of time-course gene expression profiles, it was limited to only 10 transcription factors. Thus, the computational analyses so far performed to study the innate immune response have either been limited by the size of the molecular network used, or by the lack of time-course gene expression profiles. In this study, we perform a comprehensive computational analysis of the dynamic aspects of the innate immune response in the context of a large-scale molecular network.

Several methods using condition-specific genetic, transcriptional and epigenomic data in the context of large protein-protein interaction (PPI) and protein-DNA interaction (PDI) networks have been developed, and have led to the identification of novel regulators and pathways in several cellular systems [10], [11]. These include Network Component Analysis (NCA) [12], DREM [13] and its recent update SDREM [14], ResponseNet [15] and SteinerNet [16], [17]. Data from time-course gene expression profiles is particularly informative in this context since it can capture chronological events in the cellular system. However, some of the methods listed above, like ResponseNet and SteinerNet, are insensitive to the temporal aspect of gene expression, while others like NCA and SDREM use the temporal gene expression information only to identify transcription factors activated at various time points but not to predict active networks. Others have used time-course gene expression profiles either to identify time-specific protein-modules in PPI networks [18]–[21], or to infer transcription regulatory networks activated over time [12], [13],[22]. Though all the methods described so far are relatively successful in identifying network components and modules activated at specific time points, no attempt has been made to identify paths connecting genes expressed at different time points. Such temporal paths can show potential connections between genes expressed at different stages of a response thus providing information about intermediate, transiently expressed regulators that would otherwise have been overlooked.

In this work, we studied the innate immune response in dendritic cells (DCs) stimulated by lipopolysaccharide (LPS). LPS is a component of the outer membrane of Gram-negative bacteria and specifically binds to the TLR4 receptor, triggering both the downstream MyD88 and TRIF-dependent pathways. We used time-course gene expression profiles collected at 8 time points after LPS stimulation in the context of a high-confidence PPI, PDI and post-translational modifications (PTM) network. We grouped the gene expression profiles into three groups – the initial response genes (greatest fold-change in expression between 0.5–1 hour after stimulation), the intermediate regulators (greatest fold-change in expression between 2–4 hours after stimulation) and the late effectors (greatest fold-change in expression between 6–8 hours after stimulation). We then attempted to identify the most probable paths connecting the initial response genes to the late effectors in the interaction network, while taking into account the intermediate regulators. In order to do this, we used a network flow optimization approach allowing the flow to follow a time-dependent path within the molecular network. Using this method, we were able to identify an optimal gene sub-network for activated DCs. Based on this sub-network, we identified several known core components of the innate immune response, novel down-stream participants and pathways connecting these core components. We were able to identify genes playing an important role in the innate immune response but showing no observable change in expression. We also analyzed time-course gene expression profiles of MyD88-knockout cells and TRIF-knockout cells, and compared their gene sub-networks to that obtained for wild-type DCs in order to identify the components that are independently activated in each pathway. Finally, we identified the distinct functional classes of genes expressed during different stages of the immune response and how their patterns of expression change in MyD88 and TRIF-knockout DCs compared to those in wild-type DCs.

## Results

### Optimal sub-network identification

We used a minimum cost flow optimization approach to identify important components of the innate immune response over time on LPS stimulation. A network of PPI and regulatory interactions, including transcription factor-target gene, phosphorylation, dephosphorylation and ubiquitination relationships, was prepared. Network edges were scored based on interaction reliability as obtained from the protein-protein interaction database, HitPredict [23]. Time-course gene expression levels were obtained using RNA-seq from DCs before LPS stimulation and up to 8 hours after LPS stimulation. The genes with significant changes in expression after LPS stimulation were divided into 3 groups based on the time of their greatest change in expression:

1. Initial response genes – genes showing the highest fold change in expression between 0.5–1 hour after LPS stimulation,
2. Intermediate regulators – genes showing the highest fold change in expression between 2–4 hours after LPS stimulation,
3. Late effectors – genes showing the highest fold change in expression between 6–8 hours after LPS stimulation.

**Figure 1:**
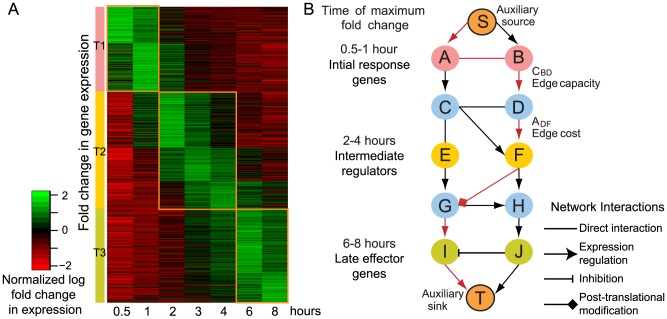
Gene expression profiles and schematic representation of the method. A. Log fold change in gene expression levels of 1047 genes showing greater than 2-fold up-regulation in dendritic cells on LPS stimulation at 7 time points. The orange blocks illustrate the partitioning of the genes into 3 groups based on the time of their highest fold change - T1: 0.5–1 hour, T2: 2–4 hours, T3: 6–8 hours, used to identify the most probable paths between them within the molecular network. B. Schematic representation of the minimum cost network flow optimization used to predict an optimal sub-network in active DCs from a large molecular network containing protein-protein interactions, protein-DNA interactions and post-translational modifications. The sub-network is obtained by optimizing the flow from the auxiliary source node (S) to the auxiliary sink node (T) such that it includes edges with the lowest edge cost, A, (highest edge reliability) and the highest edge capacity, C, (greatest fold change in expression of adjacent genes). The predicted minimum cost flow path (in red) passes through at least one of genes A and B which show altered expression between 0.5–1 hour, followed by one or more of the genes E and F with significant change in expression between 2–4 hours, before finally passing through at least one of the genes, I and J, with altered expression between 6–8 hours.

In order to identify potential paths through the molecular network connecting the genes within the three groups, we formulated the problem as a minimum cost flow optimization problem incorporating the gene expression levels in three stages. Figure 1 shows a schematic representation of the proposed method. We set our source nodes as the initial response genes. The target nodes of the network were the late effector genes. Edges of the network were assigned costs that were inversely proportional to their interaction reliability. Edges were also given a flow capacity proportional to the observed change in expression of the adjacent genes. A constraint was added to the flow optimization problem to force the flow to go through at least one intermediate regulator. We solved the optimization problem to identify the path of minimum cost for the flow to pass through the network using linear programming techniques (see Materials and methods for the problem formulation). The method found the most probable paths in the network between genes expressed in the initial response and those expressed at a later time while taking into account the genes expressed during the intermediate stage. Each edge of the optimal sub-network was assigned a flow signifying its importance. This resulted in a weighted gene sub-network where the edges were scored according to their importance. Flows were calculated for nodes, or genes, as the sum of the flows of their incoming edges. Genes with high flows were considered important due to their connection to high-flow edges. The reliability of the optimal solution was confirmed and statistical significance was calculated for each gene in the optimal sub-network by randomizing the source and target nodes (see Materials and methods). The flow assigned to a gene within the sub-network shared an inverse relationship with its statistical significance, demonstrating that a high flow was a good indicator of reliability (Figure S1). The genes with the highest flows – *Socs3*, *Nfκb1*, *Jak2*, *Jun*, *Fos*, *Cxcl10* and *Stat1* are well-known components of the innate immune response. Table 1 shows 20 genes with the highest flows in the optimal sub-network for activated wild-type DCs (See Table S1 for the list of all predicted genes and their statistical significance). As shown by the results, the method not only predicted essential genes expressed within each of the 3 groups, but also genes for which no significant change in expression was detected but were connected to others with significant changes in expression over time.

**Table 1:** Genes predicted with the highest flows in the optimal sub-network (p<0.05).

Initial response genes (0.5–1 hour)		Intermediate regulators (2–4 hours)		Late effectors (6–8 hours)		Genes with no change in expression	
Gene	Flow	Gene	Flow	Gene	Flow	Gene	Flow
Jun	13.68	Socs3	85.85	Cxcl10	10.91	Stat1	8.74
Fos	10.34	Nfκb1	76.87	Ddx58	9.33	Mapk8	8.72
Il1b	9.86	Jak2	54.44	Stat2	8.65	Irf5	7.60
Tnf	9.36	Src	38.30	Atf3	8.29	Adcy5	7.43
Cxcl2	7.59	Pik3r5	27.86	Isg15	8.15	Mapk1	7.40
Il1a	7.40	Rela	23.35	Irf7	7.30	Sp1	7.37
Akt1	6.43	Stat5a	20.40	Nos2	6.91	Stat6	7.17
Atf4	5.49	Met	18.94	Ifnar2	5.20	Sp3	7.13
Nfκbiz	5.25	Eif2ak2	17.77	Stat3	5.01	Creb1	6.88
Egr1	5.20	Irf1	17.53	Rsad2	4.97	Mapk12	6.80
Sfpi1	4.99	Ccl4	15.70	Ccl5	4.93	Il7r	6.52
Il1r2	4.79	Ccl3	15.64	Il15ra	4.13	Akt2	6.43
Traf6	4.54	Ripk2	14.43	Ifngr1	4.09	Mapk10	6.39
Nfκbia	4.39	Nfκbib	13.95	Socs1	4.07	Mapk9	6.39
Hras1	4.39	Hsp90aa1	13.94	Rac2	4.03	Rac1	6.27
Tnfaip3	4.02	Ccr2	12.58	Zbp1	4.00	Pik3r2	6.24
Ereg	4.00	Traf1	11.48	Gbp2	3.74	Ppp2ca	6.19
Vegfa	3.90	Il6	10.94	Casp1	3.31	Trp53	6.08
Areg	3.87	Ppp1cb	10.86	Cd40	3.31	Mapk11	5.62
Pgf	3.60	Icam1	10.46	Il1r1	3.27	Akt3	5.50

### Optimal sub-network evaluation

In order to evaluate the reliability of the gene network resulting from the paths identified by solving the flow optimization problem, we compared the genes in the optimal sub-network with the experimentally identified regulators of the innate immune response from previous perturbation experiments [3], [4]. Of the 125 regulators identified by Amit et al. [3], our sub-network contained 62 (49.6%), all of which had a flow greater than 1 (Table S2). In a similar study by Chevrier et al. [4], our sub-network contained 30 of the 43 known or novel regulators identified (69.8%), and 56 of the 102 (54.9%) TLR target genes affected by the perturbation of these regulators (Table S3). The sub-network also contained the gene, Polo-like kinase 2 (Plk2), which activates a distinct signaling cascade. Thus, our sub-network contained a significant number of the regulators of the innate immune response that were recently experimentally identified.

We further confirmed the quality of the predicted gene network through Gene Ontology (GO) and KEGG pathway enrichment analysis. The genes having flows greater than 1 in the sub-network, were enriched for the Toll-like receptor signaling pathway (p = 5.10e-41), Jak-STAT signaling pathway (p = 4.88e-45), pathways in cancer (p = 2.50e-41) and chemokine signaling pathway (p = 5.16e-40) among others (See Table S4 for full list). The association of the predicted genes with the innate immune response is further confirmed by the GO Biological Process terms enriched for these genes. Protein amino acid phosphorylation (p = 7.80e-36), immune response (p = 1.35e-32) and regulation of programmed cell death (p = 1.72e-29) were some of the most enriched terms (See Table S5 for full list). 49.7% of the genes identified in the optimal sub-network did not show significant change in their expression levels on LPS stimulation. In order to confirm that these genes contribute to the enrichment of functional terms associated with the innate immune response, we compared the enrichment of the KEGG pathways and the GO terms in all predicted genes with those that showed differential expression after LPS stimulation (Table S6, S7). Including predicted genes lacking differential expression significantly improved the enrichment of the KEGG pathways and the GO terms associated with the innate immune response over that observed for differentially expressed genes only. This further confirmed the association of the genes predicted in optimal sub-network with the innate immune response.

Additional analysis of GO term enrichment of genes identified in the sub-network at each time point showed the distinct processes active during different stages of the immune response. Table 2 shows the most significant GO Molecular Function and Cellular Component terms enriched in genes identified at each time point. The most significant term enriched for genes expressed between 0.5–1 hour is “transcription regulator activity” (p = 1.18e-09) for 20% of the genes indicating an upregulation of transcription factors during the first hour of the immune response. On the other hand, genes predicted at 2–4 hours are enriched for “nucleotide binding” (p = 9.33e-04, 28.5% genes) and “protein kinase activity” (p = 1.27e-03, 13% genes) suggesting a role for signal transducers. Finally, the terms enriched for genes predicted between 6–8 hours are “proteasome complex” (p = 2.98e-11, 7%) and “peptidase activity” (p = 5.2e-08, 13%) highlighting the activity of the immunoproteasome during this phase of the innate immune response. Finally, genes that were identified in the optimal sub-network but which did not show change in expression during the sampled time points were enriched for GO terms such “protein kinase activity” (p = 7.52e-31, 16%), “cytokine binding” (p = 5.9e-26, 6%) and “transcription factor activity” (p = 1.18e-07, 12%) (Table 3).

**Table 2:** GO Molecular Function and Cellular Component terms enriched in genes with significant change in expression identified in the optimal sub-network.

Time	GO Term	% genes	PValue	Bonferroni
0.5–1 hour	DNA binding	21.15	4.25E-07	1.42E-04
	Transcription regulator activity	20.38	3.54E-12	1.18E-09
	Nuclear lumen	11.53	7.32E-07	1.59E-04
	Intracellular organelle lumen	11.53	8.78E-05	1.89E-02
	Organelle lumen	11.53	9.20E-05	1.98E-02
	Transcription factor activity	10.38	1.44E-04	4.70E-02
	Cytosol	7.69	3.70E-05	7.99E-03
	Nucleoplasm	7.69	1.18E-04	2.53E-02
	Transcription factor binding	7.30	4.72E-07	1.58E-04
	Protein dimerization activity	6.92	1.96E-05	6.54E-03
	Transcription repressor activity	5.76	4.66E-06	1.56E-03
	Transcription cofactor activity	5.00	2.16E-05	7.20E-03
	MAP kinase tyrosine/serine/threonine phosphatase activity	1.92	2.42E-05	8.06E-03
	MAP kinase phosphatase activity	1.92	2.42E-05	8.06E-03
2–4 hours	Nucleotide binding	28.57	3.50E-06	9.33E-04
	Ribonucleotide binding	24.67	8.02E-06	2.14E-03
	Purine ribonucleotide binding	24.67	8.02E-06	2.14E-03
	Purine nucleotide binding	24.67	2.04E-05	5.43E-03
	ATP binding	19.48	1.56E-04	4.08E-02
	Adenyl ribonucleotide binding	19.48	1.91E-04	4.98E-02
	Protein kinase activity	12.98	4.75E-06	1.27E-03
	Protein serine/threonine kinase activity	9.74	7.52E-05	1.99E-02
6–8 hours	Cytosol	12.93	1.33E-08	2.26E-06
	Peptidase activity	12.93	1.64E-07	5.28E-05
	Peptidase activity, acting on L-amino acid peptides	11.94	1.11E-06	3.56E-04
	Endopeptidase activity	10.94	4.15E-08	1.33E-05
	Extracellular space	8.95	1.84E-04	3.07E-02
	Cell surface	7.46	2.40E-05	4.05E-03
	Proteasome complex	6.96	1.76E-13	2.98E-11
	External side of plasma membrane	5.97	4.78E-05	8.04E-03
	Cytokine binding	5.47	1.10E-07	3.51E-05
	Proteasome core complex	4.97	7.71E-13	1.30E-10
	Threonine-type peptidase activity	4.97	1.23E-12	3.96E-10
	Threonine-type endopeptidase activity	4.97	1.23E-12	3.96E-10
	Cytokine receptor activity	3.48	5.29E-05	1.69E-02
	MHC class I protein complex	2.98	1.56E-04	2.61E-02
	MHC class I peptide loading complex	1.99	1.46E-04	2.43E-02

**Table 3:** GO Molecular Function and Cellular Component terms enriched in genes with no significant differential expression identified in the optimal sub-network.

GO Term	% genes	PValue	Bonferroni
Protein kinase activity	16.29	1.39E-33	7.52E-31
Cytokine binding	6.48	1.10E-28	5.95E-26
Protein serine/threonine kinase activity	11.21	9.47E-22	5.14E-19
Cytokine receptor activity	4.55	1.15E-21	6.25E-19
Purine ribonucleotide binding	25.74	1.84E-21	9.98E-19
Ribonucleotide binding	25.74	1.84E-21	9.98E-19
Plasma membrane	32.75	4.81E-21	1.75E-18
Integrin complex	3.33	3.00E-20	1.09E-17
Purine nucleotide binding	25.92	3.35E-20	1.82E-17
Receptor complex	4.55	2.06E-17	7.49E-15
Adenyl ribonucleotide binding	20.67	2.92E-16	1.81E-13
ATP binding	20.49	3.12E-16	1.81E-13
Nucleotide binding	26.80	8.16E-16	4.22E-13
Protein tyrosine kinase activity	5.95	1.31E-15	7.23E-13
Purine nucleoside binding	21.19	1.37E-15	7.23E-13
Nucleoside binding	21.19	2.20E-15	1.21E-12
Adenyl nucleotide binding	20.84	4.57E-15	2.47E-12
Plasma membrane part	19.61	3.44E-13	1.25E-10
Transmembrane receptor protein tyrosine kinase activity	3.15	1.03E-11	5.59E-09
Cytosol	9.28	2.44E-11	8.87E-09
Transcription factor activity	11.73	2.17E-10	1.18E-07
MAP kinase activity	1.75	2.22E-10	1.21E-07
Cell surface	5.78	1.84E-08	6.70E-06
Chemokine receptor activity	1.75	4.22E-08	2.29E-05
Growth factor binding	2.80	5.01E-08	2.72E-05
Transcription regulator activity	14.54	5.23E-08	2.84E-05
Chemokine binding	1.75	6.70E-08	3.64E-05
Protein phosphatase type 2A complex	1.40	7.77E-08	2.83E-05
Nucleoplasm	8.41	9.65E-08	3.51E-05
GTPase activity	3.50	2.64E-07	1.43E-04

To check the quality of the network paths predicted by the method, we identified all the possible paths predicted in the optimal sub-network that matched a directed path of the same length in a KEGG pathway. Our method was able to predict directed paths of 3 edges or more in 13 KEGG pathways, including the Jak-STAT signaling pathway, the Chemokine signaling pathway, the Toll-like receptor pathway and the MAPK signaling pathway (Table 4, Table S8). The longest predicted directed path contained 7 edges and was part of the Jak-STAT signaling pathway. Thus, the method was able to partially recover known pathways in the form of short paths connecting genes expressed at consecutive time points. We also identified all shortest paths up to 3 edges (i.e. containing 4 nodes at most) between genes expressed at different stages of the immune response and checked how well they were represented in the same KEGG pathway. We found that 84.9% of the predicted paths have at least 2 genes in the same KEGG pathway, while 11.6% of the paths have all genes in the same KEGG pathway (Figure S2). Taken together, these results confirm the reliability of the optimal gene sub-network identified for activated wild-type DCs.

**Table 4:** Directed paths predicted in the optimal sub-network found in KEGG Pathways.

KEGG Pathway	Edges in longest predicted path
Jak-STAT signaling pathway	7
Chemokine signaling pathway	5
Cell cycle	4
Complement and coagulation cascades	4
MAPK signaling pathway	3
Axon guidance	3
Toll-like receptor signaling pathway	3
Tuberculosis	3
Focal adhesion	3
ErbB signaling pathway	3
Adherens junction	3
Gap junction	3
GnRH signaling pathway	3

To demonstrate the utility of our algorithm, we compared the optimal sub-network identified by our method to that identified using a non-temporal minimum cost flow optimization method, ResponseNet [15]. Using minimum cost flow optimization through our initial network, ResponseNet identified paths from the initial response genes to the late effectors without taking the intermediate regulators into account (Table S9). Table 5 shows the results of the comparison between the optimal sub-networks predicted by our method and ResponseNet. ResponseNet identified fewer genes and interactions in the predicted sub-network. More significantly, since there was no constraint for the flow to pass through the intermediate regulators, it identified only 49 of these as compared to the 154 by the current method. Our method also identified significantly higher number of known regulators in the innate immune response in addition to longer paths in associated pathways. On the other hand, ResponseNet failed to identify a directed path of 3 or more edges within any KEGG pathway associated with the innate immune response. These results clearly demonstrate that including the intermediate regulators into the problem formulation, as we propose here, improves the ability of the method to predict candidate genes and associated networks using time-course gene expression profiles.

**Table 5:** Components of the optimal sub-network predicted by the proposed method and ResponseNet.

Method	Predicted Genes	Predicted Edges	Intermediate regulators in network	Edges to Intermediate regulators	Amit et. al regulators predicted	Chevrier et al. regulators predicted	Chevrier et al. TLR targets predicted#	KEGG pathways with predicted paths (maximum length*)
**ResponseNet**	848	1080	49	147	39.2%	53.5%	39.2%	0 (3 edges)
**This method**	1224	2242	154	1179	49.6%	69.8%	54.9%	13 (7 edges)

# one gene with 0.05<p<0.1.

* length indicates the maximum number of consecutive directed edges identified in the pathway.

### Identified genes and their associated networks

**Figure 2:**
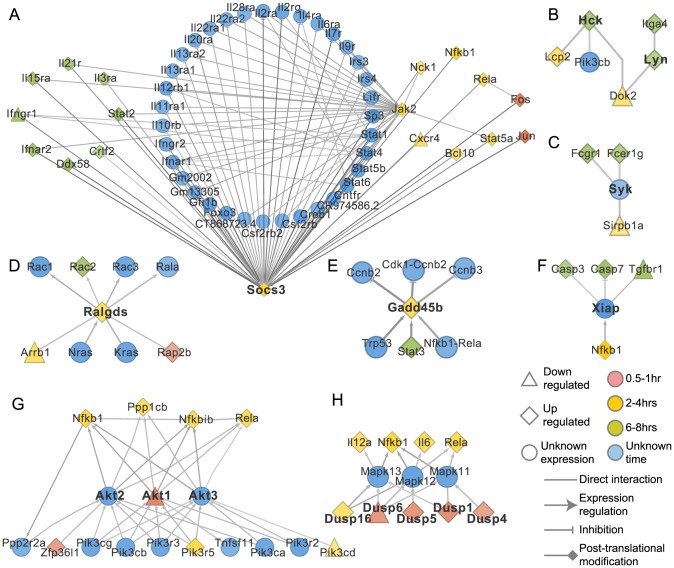
Associated interactions for selected proteins within the optimal sub-network. The sub-networks show the most probable associations of some of the genes/proteins within the 3 time-based groups. Several genes showing no change in expression are also part of these sub-networks. A. Socs3, B. Hck and Lyn, C. Syk, D. Ralgds, E. Gadd45b, F. Xiap, G. Akt family of proteins, H. Dusp proteins. Edge and node colors indicate relative flow with darker colors signifying greater flow.

The gene predicted with the highest flow in the optimal sub-network was Suppressor of cytokine signaling 3 (*Socs3*) followed by Nuclear factor κb1 (*Nfκb1*). Both genes were significantly upregulated between 2–4 hours and are well-known regulators of the innate immune response. Socs3, along with Socs1 and Socs2, is an inhibitor of cytokine signaling pathways. It is a key regulator of interleukins 6 and 10 (Il6 and Il10) [24]. In the identified sub-network, *Socs3* is induced by the primary regulators of the immune response such as Nfκb1 and inhibits a large number of proteins, specifically interleukin receptors (Figure 2a).

*Nfκb1* is induced both in the early and late phase of the innate immune response and is primarily responsible for the expression of inflammatory cytokines. Other genes identified with high flows were the Janus kinase 2 (*Jak2*), Rous sarcoma oncogene (*Src*) and phosphoinositide-3-kinase, regulatory subunit 5 (*Pik3r5*), all of which have been implicated in the TLR response pathway. *Src*, a protein tyrosine kinase that modulates a large number of signaling pathways during the innate immune response, was upregulated between 2–4 hours. Along with *Src*, other tyrosine kinases from the Src family, such as *Hck* and *Lyn*, were also identified (Figure 2b). *Syk*, another protein tyrosine kinase of the Syk-ZAP70 family that is found in innate immune cell types, was also identified as part of the network though no significant change in gene expression levels was detected at the tested time points (Figure 2c). Several other components of the Src signaling pathways like Card9, Cblb, Fcerγ and various integrins were also identified within the gene sub-network.

Among other known regulators, the induction of *Ralgds* by Ras proteins, and the further upregulation of the *Rac* genes, was also detected (Figure 2d). Gadd45b, an anti-apoptotic inhibitor induced by Nfκb [25] was also part of the sub-network. *Gadd45b* was significantly upregulated between 2–4 hours and was predicted to inhibit the cyclins B2, B3 and CDK (Figure 2e). Another anti-apoptotic inhibitor, the X-linked inhibitor of apoptosis (*Xiap*) was also identified. Xiap is regulated by Nfκb and in turn inhibits Casp3 and Casp7 thus controlling apoptosis (Figure 2f) [25].

Another class of proteins identified, were the Akt serine-threonine protein kinases Akt1, Akt2 and Akt3, which are downstream effectors of the PI3K pathway (Figure 2g). Expression level change was only observed for *Akt1* which was down-regulated at 0.5–1 hours followed by an up-regulation at 3 hours. Other predicted components include the Dual specificity phosphatases (DUSP proteins) which were significantly upregulated between 0.5–1 hour, except *Dusp6*. The Dusp proteins regulate the immune response by dephosphorylating the Map kinases and repressing the LPS-induced inflammatory response (Figure 2h). Interestingly, the network indicated that the *Dusp* genes were expressed within the early stages of the innate immune response suggesting that control of inflammation begins soon after its induction.

**Figure 3:**
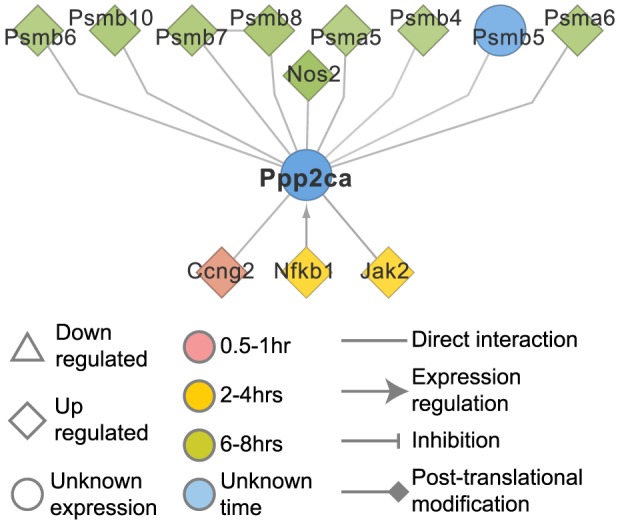
Ppp2ca and its potential role in immunoproteasome regulation. The sub-network shows the most probable associations of ppp2ca that are active during the innate immune response. Pppc2a is induced by Nfκb1 and interacts with several immunoproteasome subunits. Edge and node colors indicate predicted flow with darker colors signifying greater flow.

Many of the genes identified in the network do not show any significant change in expression after activation of the DCs, but are known to be essential for the response. An example is the protein phosphatase 2a catalytic subunit α (p*pp2ca*) which has a high flow in the sub-network. A serine threonine phosphatase required for the dephosphorylation of the 20S proteasome subunits, ppp2ca is known to affect the ability of the proteasome to degrade substrates, along with protein kinase A (PKA) [26]. Ppp2ca has also been recently shown to play an important role in the regulation of endotoxin tolerance through the regulation of MyD88 activity [27]. The identified gene sub-network indicated extensive interactions between ppp2ca and the subunits of the immunoproteasome, suggesting a role of ppp2ca in the regulation of the immunoproteasome (Figure 3). The immunoproteasome is induced by interferons and is central to the regulation of the immune response and in the prevention of auto-inflammatory diseases through its ability to degrade toxic protein aggregates during cytokine-induced oxidative stress [28].

### Analysis of MyD88 and TRIF-knockout dendritic cells

We applied the method described above to time-course gene expression profiles obtained from DCs of MyD88 and TRIF-knockout mice in the context of the comprehensive molecular interaction network. MyD88 and TRIF are essential components of the innate immune response and trigger distinct pathways that result in the activation of early and late phase Nfκb, respectively.

Previous studies have shown that Nfκb and Mapk8 (JNK) are activated in a delayed manner in MyD88-knockout cells. However, inflammatory cytokines like IL12 or TNFα are not produced [29]. In order to identify the MyD88-independent response network, we used gene expression levels from MyD88-knockout DCs to assign edge capacities, and removed the MyD88 gene and its links within the network prior to solving the minimum cost flow optimization problem (See Table S10 for identified genes and edges). We performed a similar analysis on the data from TRIF-knockout DCs by removing TRIF and its links from the network and predicting a MyD88-dependent response network on LPS stimulation (See Table S11 for identified genes and edges).

**Figure 4:**
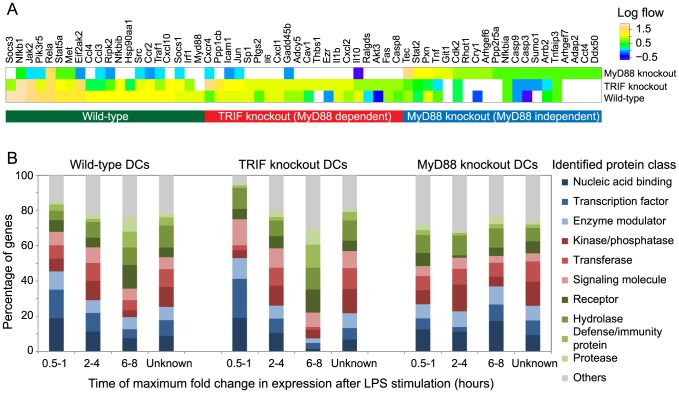
Comparison of MyD88 and TRIF-knockout gene sub-networks. A. Comparison of the predicted flows of 20 genes from the optimal sub-networks of wild-type, MyD88-knockout and TRIF-knockout DCs. The heat map shows the similarity between the wild-type and the TRIF-knockout response to LPS stimulation through the large number of common genes predicted with high flows. B. Functional classes of genes based on the time of their highest fold change in expression and identified in the optimal sub-networks identified for wild-type, TRIF-knockout and MyD88 DCs on activation by LPS. “Unknown” denotes predicted genes that do not show significant change in expression on activation. Distinct gene groups are activated at different time points during the innate immune response. Protein class “Others” includes cytoskeletal proteins, cell adhesion molecules, calcium-binding proteins, ligases, transfer-carrier proteins, oxidoreductases, extracellular matrix proteins, transporters, chaperones, structural proteins, membrane traffic proteins, transmembrane receptor regulators, lyases, isomerases, cell junction proteins, surfactants and storage proteins.

A comparison of the genes and their flows in the identified sub-networks suggests that the response pathways active in the wild-type and TRIF-knockout sample are similar (Figure 4a). The active sub-networks identified for both these samples are enriched in the KEGG pathways “Cytokine-cytokine receptor interaction” (p = 1.13e-29), “Jak-STAT signaling pathway” (p = 2.34e-15) and “Toll-like receptor signaling pathway” (p = 6.07e-11). These findings suggest the dominance of the MyD88 pathway in the wild-type response. Indeed, this dominance has been previously observed during pulmonary infection [30]. On the other hand, the most enriched pathways in the genes exclusively identified in the MyD88-knockout network are the “Circadian rhythm” (p = 6.29e-5) and “Ubiquitin mediated proteolysis” (p = 3.2e-4) suggesting an association between these pathways and the MyD88-independent, TRIF-dependent pathway (Table 6, Tables S12 and S13).

**Table 6:** KEGG pathways enriched in genes identified exclusively in MyD88 and TRIF-knockout sub-networks.

Sample	Term	% genes	PValue	Bonferroni
	Circadian rhythm	1.67	4.92E-07	6.29E-05
	Small cell lung cancer	3.56	9.38E-07	1.20E-04
MyD88-knockout	Ubiquitin mediated proteolysis	4.39	2.50E-06	3.20E-04
	Pathways in cancer	7.32	2.59E-06	3.32E-04
	Alzheimer's disease	4.39	1.89E-04	2.39E-02
	Focal adhesion	4.60	2.16E-04	2.72E-02
	Cytokine-cytokine receptor interaction	15.45	9.39E-32	1.13E-29
	Jak-STAT signaling pathway	9.09	1.95E-17	2.34E-15
	MAPK signaling pathway	11.59	5.36E-16	6.66E-14
	Chemokine signaling pathway	8.86	8.70E-14	1.04E-11
	Toll-like receptor signaling pathway	6.36	5.05E-13	6.07E-11
	NOD-like receptor signaling pathway	4.55	1.77E-10	2.12E-08
	Focal adhesion	7.95	5.65E-10	6.78E-08
	GnRH signaling pathway	5.00	2.06E-08	2.47E-06
	Progesterone-mediated oocyte maturation	4.32	3.10E-07	3.72E-05
	Apoptosis	4.32	4.49E-07	5.39E-05
	Pathways in cancer	8.86	1.99E-06	2.38E-04
TRIF-knockout	Prion diseases	2.50	8.44E-06	1.01E-03
	T cell receptor signaling pathway	4.32	4.07E-05	4.87E-03
	Leukocyte transendothelial migration	4.32	4.57E-05	5.47E-03
	Vascular smooth muscle contraction	4.32	5.12E-05	6.13E-03
	Pancreatic cancer	3.18	8.37E-05	1.00E-02
	Hematopoietic cell lineage	3.41	1.11E-04	1.33E-02
	Gap junction	3.41	1.45E-04	1.72E-02
	Neurotrophin signaling pathway	4.32	1.49E-04	1.77E-02
	Natural killer cell mediated cytotoxicity	4.09	2.09E-04	2.48E-02
	Melanogenesis	3.64	2.17E-04	2.57E-02
	Long-term potentiation	2.95	2.61E-04	3.09E-02
	Bladder cancer	2.27	2.76E-04	3.26E-02
	Insulin signaling pathway	4.32	3.18E-04	3.75E-02

In order to identify the dominant changes in the immune response over time, we classified the genes from the optimal sub-networks obtained for the wild-type, MyD88-knockout and TRIF-knockout DCs into functional classes. Global changes in the expression patterns of genes identified as part of the optimal sub-network at each of the 3 stages showed a dominance of functionally distinct groups at different times during the immune response (Figure 4b). In wild-type DCs, transcription factors and enzyme modulators were predominantly expressed during 0.5–1 hour after LPS stimulation. On the other hand, kinases and signaling molecules were abundant between 2–4 hours after stimulation. Finally, proteases and defence/immunity proteins along with receptors showed the greatest changes in expression in the late phase of the immune response between 6–8 hours. TRIF-knockout DCs showed similar changes in the expression patterns of genes. However, these patterns were significantly different in the MyD88-knockout DCs. Transcription factors were not as significantly upregulated in the early phase, but more so in the late phase, when the expression of proteases and defence/immunity genes was significantly reduced. Thus, the identified sub-networks suggest a pattern in the global change in gene expression during the different stages of the immune response. The similarity of the patterns of gene expression in the TRIF-knockout DCs and wild-type DCs further support the dominant role of the MyD88-dependent pathway in the innate immune response. An analysis of the functional distribution of the genes predicted in the network, but not showing significant differential expression on activation, illustrates their similarity to the intermediate regulators in the wild-type as well as knockout DCs.

Several important components of the innate immune response were identified in both knockout sub-networks, however, with significantly different flows. *Nfκb1*, *Jak2* and *Socs1* were genes with the highest flows (>40) in the TRIF-knockout network. These genes were also identified in the MyD88-knockout network, but with flows just above 1. This disparity in the flows possibly indicates their changing levels of expression and significance within the two sub-networks. The sub-network associated with MyD88-knockout DCs had different genes with high flows – *Akt3*, *Casp8* and *Stat2*. Interestingly, the kinase Pik3r5 had similar levels of predicted flow in both knockout networks. It was upregulated in both instances but much more so in the MyD88-knockout DCs.

**Figure 5:**
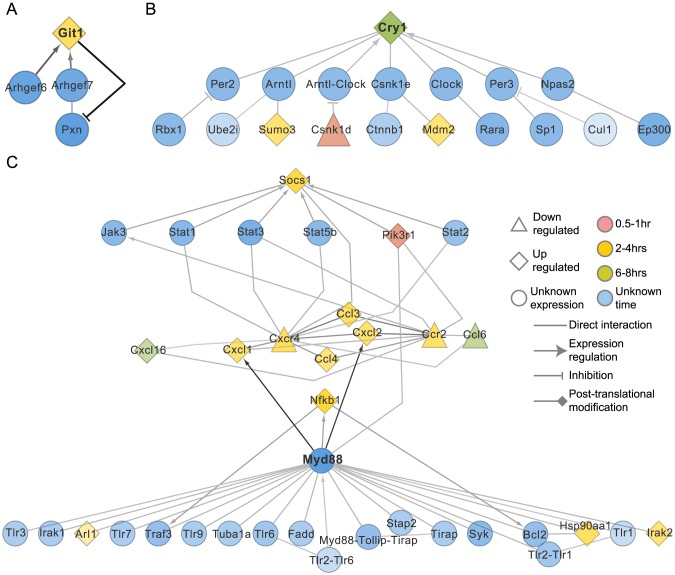
Interactions associated with genes identified in MyD88-knockout and TRIF-knockout response. Genes predicted to be part of the optimal sub-network in either the MyD88-knockout or the TRIF-knockout samples. A. Sub-network associated with Git1 which has the highest flow in the MyD88-knockout sample. B.Cry1, a component of the circadian complex and its associated network. C. A sub-network identified for the TRIF-knockout DCs showing the role of MyD88, Nfκb, the cytokines, Jak-Stat pathway and finally Socs1 induction. Edge and node colors indicate relative flow with darker colors signifying greater flow.

Git1 and Cry1 were two of the important candidates identified only in the MyD88-knockout gene network. Git1 (G-protein coupled receptor kinase interacting protein 1) acts in the formation of a scaffold to bring together molecules to form signaling modules and increase the speed of cell migration. Its role in the innate immune response is currently not known. However, it was significantly upregulated in the MyD88-knockout sample and found to interact with Pxn, Arhgef6 and Arhgef7 (Figure 5a).

The other important gene identified, *Cry1*, is a key component of the circadian core oscillator complex. The role of Cry1 in the negative regulation of the activation of Nfκb and further induction of proinflammatory cytokines has been recently elucidated [31]. *Cry1* was significantly upregulated in the MyD88-knockout DCs between 6–8 hours after stimulation and could potentially be regulating the activation of Nfκb signaling. Though *Cry1* was part of the gene network associated with the activation of wild-type DCs, it was not identified in the optimal gene sub-network associated with TRIF-knockout DCs, suggesting that the upregulation of *Cry1* and its role might be controlled by the MyD88-independent, TRIF-dependent pathway (Figure 5b).

The MyD88-knockout associated gene network also contained a number of genes from the E2 and E3 ubiquitin-conjugating enzyme families, including several members of the Trim family, which are known for their role in suppressing the immune response by increasing the ubiquitination and subsequent degradation of regulatory genes [32]. The selective prediction of these ligases in the MyD88-knockout response network suggests that proteolytic degradation might also be predominantly affected by the TRIF-dependent pathway.

The response network identified for the TRIF-knockout sample highlights the wild-type MyD88 pathway wherein MyD88 triggers the activation of Nfκb which in turn induces the inflammatory cytokines, further inducing the *Jaks* and *Stats* and finally upregulating the *Socs* genes which repress the inflammatory response (Figure 5c).

## Discussion

We used a method based on minimum cost flow optimization to identify paths connecting genes expressed during 3 major stages of the innate immune response within a large molecular interaction network. This method was able to identify a sub-network active during the innate immune response, with genes and interactions associated with flows corresponding to their importance in the network, while taking their time of expression into account. A large number of genes were identified in spite of their lack of significant change in expression, but based on how well they were connected to genes that showed significant changes in expression over time.

The optimal sub-network identified in this study is based on gene expression profiles obtained from LPS stimulated DCs and represents a pathogen-specific response of the innate immune system against infection by Gram-negative bacteria. A significant number of previously known components of the innate immune response were identified along with important pathways triggered immediately after LPS stimulation in DCs. One of the genes identified was the protein phosphatase 2 catalytic subunit α (*pppc2a*), recently found to be an important player in the immune response [27]. Based on the interactions of this protein in the optimal sub-network, we propose an additional role for this protein in the regulation of protein degradation by the immunoproteasome. The differences between the MyD88 and TRIF-dependent pathways are difficult to predict based on the wild-type response network alone due to the large overlap between these two pathways. Both pathways result in the activation of Nfκb and its downstream effectors. However, the analyses of the activated MyD88 and TRIF-knockout DCs performed here helped clarify their difference. The results indicate the dominance of the MyD88-dependent pathway during the innate immune response and the association of the TRIF-dependent pathway with the circadian genes and those involved in immunoproteasomal degradation. Both these findings need to be investigated further.

The wild-type response sub-network also shows the distinct functions of genes expressed during the three stages of the innate immune response. The enrichment of transcription factors during the early stage highlights the induction of the immune response. This is followed by significant changes in the expression of kinases and signaling molecules activating the signaling cascades during the intermediate stage. These in turn lead to the expression of defense/immunity proteins, such as cytokines in the late phase of the immune response. The late phase is also characterized by an increase in the expression of proteases signifying the start of suppression of the immune response through the degradation of proteins promoting inflammation.

There are currently very few methods available that allow the use of time-course gene expression profiles for the prediction of active gene sub-networks. Two such methods, NCA and SDREM, use the temporal gene expression information only to identify transcription factors activated at various time points but not to predict the gene sub-networks. Additionally, SDREM requires source genes to be defined based on prior knowledge of the pathway and is very slow. Our method allows the use of time-course gene expression profiles and attempts to identify optimal paths between genes expressed at subsequent stages of a cellular response over time. Due to the use of connectivity as additional evidence, the method proposed here has a limited dependence on gene expression levels, thus identifying several components lacking significant changes in expression on LPS stimulation. Additionally, important regulators were identified from the genes showing changes in expression levels based on their connections within the network, thus limiting the effect of erroneous experimental observations. The approach proposed was used to identify time-dependent gene sub-networks in activated immune cells. However, this method is independent of the system studied and can be used in any other biological system that changes over time, such as embryonic development or cellular response to stress.

The method proposed here is based on minimum-cost flow optimization approach through a large interaction network. A variation of the method, ResponseNet, has been previously used in yeast to identify optimal paths within a yeast molecular network leading from genetic hits to differentially expressed genes without accounting for transcriptional changes over time [15]. Our method differs from ResponseNet in its ability to analyze time-course gene expression profiles. The source nodes and targets of the flow optimization problem are both differentially expressed genes. Most importantly, our method has an additional constraint that forces the predicted flow through genes showing significant differential expression at intermediate time points. This constraint greatly improves the prediction performance of the minimum cost flow optimization by identifying a greater number of known regulators and associated pathways. Additionally, the reliability scores used to weight the network edges are derived from the genomic features and functional annotations of the interacting proteins rather than the characteristics of the experiments in which they were identified [33]. One of the advantages of the original method was the identification of genes whose change in transcriptional activity cannot be detected in expression detection experiments. In addition to this, our method also has the ability to identify intermediate regulators acting between different stages of the response. Further, the method proposed here succeeds in capturing sections of KEGG pathways and several known candidate genes associated with the innate immune response.

The method described in this study requires that time-course gene expression profiles from a biological system be partitioned into three stages – early, intermediate and late. While this partitioning works reasonably well for the innate immune system, it may not necessarily be possible for other biological systems. Additionally, it is likely that grouping of time points potentially hides certain relationships between genes resulting in a network that is not completely representative of cellular processes. Extending the method to include additional time points would improve the quality of the sub-network predicted. The inclusion of more interactions and pathway information would further increase the probability of identifying novel candidate genes.

A problem common to all such methods that attempt to predict pathways using gene expression data is the difficulty in completely reconstituting existing pathways on the basis of changes in gene expression levels alone. This is because genes are not necessarily expressed in the order of their known role in a pathway (Figure S3, S4, S5, S6, S7, S8). This problem can be partially addressed by including data about protein levels and post-translational modification events. An associated problem is the dependence of the prediction accuracy on the frequency at which gene expression levels are monitored. The currently prevalent time intervals of 30 minutes and 1–2 hours after stimulation do not accurately represent the time-scale of cellular events which take place on the scale of seconds to minutes [34]. This is illustrated by the fact that the most important regulatory gene, Nfκb1, showed high levels of expression at the first time point – 0.5 hours, indicating that the expression data used here does not include a significant number of events that occur between 0 and 0.5 hours. The emergence of Socs3 as a more important component of the optimal sub-network than Nfκb1 might also be a result of the experiment focusing not on the TLR pathway, but events that follow after the first effectors have already been expressed i.e. Nfκb signaling pathway, chemokine-chemokine signaling pathway, etc. Thus, experiments that monitor gene expression levels starting immediately after activation and at frequent time intervals would help improve the accuracy of the predicted network.

Despite these drawbacks, our results clearly demonstrate that the method described here is capable of predicting active gene sub-networks from time-course gene expression profiles with reasonable accuracy.

### Conclusions

The innate immune response is complex and occurs through multiple pathways. The interplay within the activated pathways makes the identification of novel components and their associations difficult. In this study, we addressed this issue by using time-course gene expression profiles of activated dendritic cells in combination with a comprehensive molecular interaction network. We developed a method based on minimum cost flow optimization in a large interaction network to identify paths between genes expressed at different time points of the immune response. Using this method, we identified an optimal gene sub-network activated during the innate immune response. We confirmed the role of several known and novel components in the identified network and suggest a role for the protein ppp2ca in the regulation of the immunoproteasome. A flow value was assigned to each identified gene and interaction within the network indicative of its importance. We also compared the response of the wild-type DCs with DCs from MyD88-knockout mice and TRIF-knockout mice and identified the global changes in expression patterns of genes in distinct functional classes. Our results are consistent with previous studies suggesting the dominant role of the MyD88-dependent pathway. We further showed that genes related to proteasomal degradation and circadian rhythms are primarily associated with the MyD88-independent, TRIF-dependent pathway. The method proposed here is independent of the biological system and can be used to identify time-dependent gene sub-networks with the help of time-course gene expression profiles related to any other cellular conditions. Future work in this area will be aimed at developing methods to accurately predict longer pathways while incorporating time-course gene expression profiles from multiple time points without the necessity of grouping them.

## Materials and Methods

### Samples and time-series experiments

GM-CSF-induced bone marrow-derived dendritic cells (GM-DCs) were prepared from C57BL6/J mice (purchased from Japan Clea Inc.) as described previously [3]. The cells were stimulated with LPS from *Salmonella minessota* Re-595 (purchased from Sigma) at a concentration of 100 ng/ml. Stimulated cells were harvested at 0, 0.5, 1, 2, 3, 4, 6, 8, 16, 24 hours after stimulation. Total RNA was extracted from the cells using TRIzol (Invitrogen) according to the manufacturer's instruction. The RNA was subjected to RNA-seq as described in a previous study [35]. Mice deficient in MyD88 or TRIF were prepared as described in an earlier study [36], [37]. The RNA-seq data is available in the Sequence Read Archive under the accession number DRA001131.

### Mapping and expression level identification

The RNA-Seq data for the wild type, MyD88-knockout and TRIF-knockout DCs at 10 time points, from 0 to 24 hours after LPS stimulation, were obtained in the form of 35 bp single-end reads. The reads were mapped to the RefSeq mm9 mouse reference genome [38] using Bowtie [39]. Exon-exon junctions were found using TopHat [40] with each read having at most 2 mismatches and 20 mappings to the reference genome, and a minimum intron length of 70 bp. For each read, the mapping with the highest alignment score was selected. The mapping statistics are shown in Table S14. Transcript abundances for all three samples at 10 time points were estimated using Cufflinks and Cuffdiff [41] using the –T option to treat the samples as a time-series. The data from the last two time points, 16 hours and 24 hours, was not used in this study because we were concerned about the effect of their large separation from the prior time points on the quality of the sub-network predicted. Maximum absolute log fold change in expression was calculated for each gene over all time points, as follows:
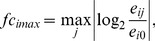
(1)Where 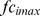 = maximum absolute log fold change for gene *i* over time *j* where *j* = {0.5,1,2,3,4,6,8},

*e_ij_* = transcript abundance of gene *i* at time *j* hours after LPS stimulation,
*e_i0_* = transcript abundance of gene *i* at time *0* hours before LPS stimulation.

Genes with at least 2 fpkm in 50% of the experiments, at least 10 fpkm for 2 or more time points and an absolute fold change greater than 2 for at least one time point in each sample were considered for further analysis. Each selected gene was assigned to one of the following groups depending on the time at which it showed the highest absolute fold change (Figure 1A):

1. Initial response genes – highest absolute log fold change between 0.5–1 hour after LPS stimulation
2. Intermediate regulators – highest absolute log fold change between 2–4 hours after LPS stimulation
3. Late effectors – highest absolute log fold change between 6–8 hours after LPS stimulation

The genes and their expression levels are shown in Tables S15, S16, S17.

### Network preparation

A network of regulatory and physical interactions from mouse was prepared by combining the following datasets:

1. Protein-protein interactions (PPIs): High confidence mouse PPIs were obtained from HitPredict (likelihood>1). HitPredict is a database of PPIs combined from multiple sources and scored for their reliability based on the genomic features of the interacting proteins [23], [33]. Core mouse interactions related to the immune response were taken from Innatedb [42]. Further, high confidence PPIs for human proteins were taken from HitPredict and their mouse orthologs were identified using Homologene [38].
2. Transcription regulatory data: Transcription factor – target gene relationships from TRANSFAC [43] were added to the network.
3. Pathways from KEGG: All functional associations, with the exception of “missing interactions”, from KEGG pathways [44], [45] were added to the network.

Table S18 shows the counts of the different interaction types included in the network. PPIs were considered as bi-directional edges whereas all other associations (transcription factor–target gene, functional association, expression regulation, post-translational modification and inhibition) were considered uni-directional. Genes and their corresponding proteins were represented by a single node in the network.

### Network edge scores

The edges of the network were weighted according to their reliability. Reliability scores provided by HitPredict and TRANSFAC were used. Innatedb core PPIs and interactions from KEGG pathways were uniformly assigned a high reliability score of 999 since these were manually curated. All scores were scaled to values between 0 and 0.8 as shown in Table S19.
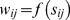
(2)Where 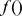 = scaling function
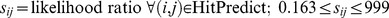


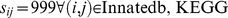


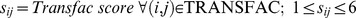
The complete network of 103218 interactions among 12856 proteins, or protein complexes, along with the data source, reliability scores and edge weights is given in Table S20.

### Linear programming formulation

The network was denoted by a graph G = (V, E) with E edges and V nodes (including the auxiliary source S and the auxiliary sink T). The auxiliary source, S, was connected to the set of initial response genes (*G_T1_*), while the auxiliary sink, T, was connected to the late effector genes (*G_T3_*). Direct edges between *G_T1_* and *G_T3_* were excluded. The intermediate regulators (*G_T2_*) were also a part of the network but not connected to the S or T nodes. All edges, E, were assigned a capacity and a cost (See Figure 1B).

#### Edge capacities

The capacity of an edge specifies the maximum flow that can pass through it. The edge capacities differ with the expression levels of genes to which they are connected. The edge capacities are defined as follows:

For edges between the auxiliary source, S, and the initial response genes *G_T1_*,
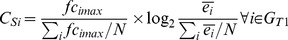
(3)For edges connected to the intermediate regulators *G_T2_*,
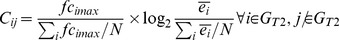
(4)

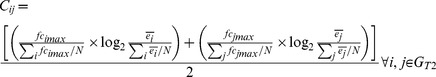
(5)For edges between the late effectors, *G_T3_*, and the auxiliary sink T,
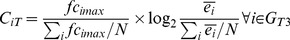
(6)For all other edges, not connected to the intermediate regulators or the auxiliary source and sink,
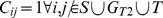
(7)

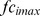
 = maximum absolute log fold change of gene *i*, as per equation (1)

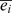
 = average expression level of gene *i* in fpkm across all time points considered,
N = number of genes with significant change in expression,
S = auxiliary source node, T = auxiliary sink node,
*G_T1_* = genes showing maximum absolute fold change between 0.5–1 hour,
*G_T2_* = genes showing maximum absolute fold change between 2–4 hours,
*G_T3_* = genes showing maximum absolute fold change between 6–8 hours.

In equations (3)–(6) above, the first term takes into account the relative fold change of the gene allowing for greater edge capacity with greater fold change. Since RNA-seq data was used and transcripts with greater fragment counts are considered more reliable, the absolute fragment counts were taken into account by the second term when calculating the capacity. The capacity of the incoming and outgoing edges connected to *G_T2_* genes was calculated using only the relative fold change of the gene and its fragment count, while not considering those of adjacent *G_T1_* and *G_T3_* genes. This ensured that the selection of the optimal path was affected by the change in expression of the *G_T2_* genes only. The fold change in expression and average fragment count of *G_T1_* and *G_T3_* genes was used to assign capacities only to the edges that connected them to the auxiliary source and the auxiliary sink, respectively. Thus, the source and target genes selected as the starting and end points of the optimal paths were dependent upon the fold changes in expression levels of the *G_T1_* and *G_T3_* genes only and did not affect the subsequent edges selected.

#### Edge costs

For edges connected to genes in one of the three stages, the weights were adjusted to correspond to their capacities as follows:
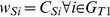
(8)

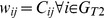
(9)

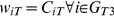
(10)

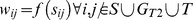
, as per equation (2)

The edge costs were calculated as:
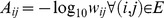
(11)


#### Problem formulation

The goal of the problem formulation was to identify paths from auxiliary source, S, to auxiliary sink, T, that minimized the cost of the flow through the network while passing from the initial response genes (*G_T1_*) to the late effectors (*G_T3_*) through the intermediate regulators (*G_T2_*). In order to optimize the flow, 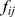, from nodes *i* to *j*, we consider the following optimization problem:
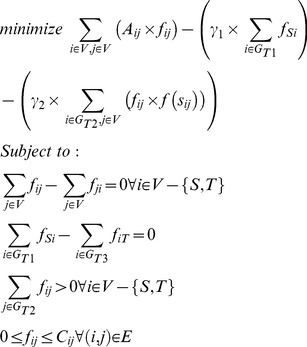
(12)

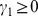
, is a constant to adjust the number of *G_T1_* genes included in the optimal sub-network,

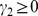
, is a constant to adjust the number of *G_T2_* genes included in the optimal sub-network,

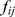
 = flow between nodes *i* and *j*,

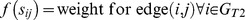
, as defined by equation (2).

This problem formulation differs from a previous variation of this method [15] in three significant aspects – 1) the start and end points of the paths to be identified are genes with expression changes at different time points in a response, 2) the additional tuning factor 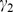, scaled by the original edge reliability 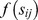, which allows control over the capacities of the edges connected to the intermediate regulatory genes (*G_T2_*), and 3) the added constraint denoted by equation (12) which forces the flow to pass through at least one intermediate regulatory gene. The optimization problem was solved using the GNU Linear Programming Kit. The solution to this problem identified the most probable set of edges connecting genes with large changes in expression from each of the three time-dependent groups.

We solved the optimization problem for all combinations of 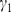 and 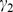 ranging from 0 to 5 in intervals of 0.5 (Table S21). The optimal sub-network with the highest number of source nodes and less than 1% unreliable edges (edge weight<0.5) was chosen with 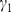 and 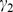 values of 0.5 and 0 respectively. The predicted nodes were assigned a flow by combining the flows of all incoming edges to each node. Nodes with higher flows were considered important in the innate immune response.

The stability of the optimal sub-network was tested using the method described in Mahadevan and Schilling [46], and used in Yeger-Lotem et al. [15]. Briefly, we fixed the optimization score to that obtained for the above optimal sub-network, and alternately maximized and minimized the flow for each edge in it to identify the bounds of the alternate optimal solutions for this problem. 97% of the nodes and 93% of the edges from the optimal sub-network were unchanged in the 4466 alternate optimal solutions obtained (median values). The median change in predicted flow for the nodes and the edges in the alternate solutions was 0. These results indicated that the alternate solutions to this optimization problem did not differ significantly from the presented sub-network and further support its reliability.

### Statistical evaluation

The genes identified as part of the optimal gene sub-network were assigned a statistical significance. This was done by solving the minimum cost flow optimization problem 5000 times using the original molecular interaction network but with randomly selected genes in the *G_T1_*, *G_T2_* and *G_T3_* sets i.e. initial response genes, intermediate regulators and late effectors in numbers equal to those from the real sample. The p-value was calculated as the fraction of solutions in which a gene was identified with an equal or higher flow than that in the optimal sub-network and with at least all the connecting edges in the optimal sub-network. We observed that the predicted flow in the final network increased with decreasing p-value (Figure S1) suggesting that a high flow was a good indicator of high statistical significance and hence greater reliability.

### ResponseNet implementation

The ResponseNet algorithm was implemented as a non-temporal minimum cost flow optimization method. The problem formulation was changed to remove the constraint in equation (12) thus allowing the flow to go from the initial response genes (source nodes) to the late effectors (target nodes) without being constrained to pass the intermediate regulators. Additionally, the term involving 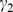 was also removed from the optimization problem. The algorithm was run on the same network as our method with identical edge costs. The capacities of edges *G_T1_ – S* and *G_T3_ – T* were set as described in equations (3) and (6). The capacity of all other edges was set to 1. The optimal solution was calculated for 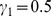 and the identified genes were compared to known regulators and KEGG pathways as described in the Results.

### Path prediction

All possible paths within the optimal sub-network from the initial response genes (*G_T1_*) to the late effectors (*G_T3_*) were identified and compared to all KEGG pathways to determine their overlap.

Paths were predicted between genes in the groups *G_T1_*, *G_T2_* and *G_T3_* by finding the weighted shortest paths [47] of up to 3 edges in the optimal sub-network. The edges were weighted as per the formula suggested by Opshal et al. [48]:
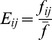
(13)where 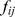 = flow assigned to edge *(i, j)*,

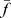
 = average flow of all edges in the optimal sub-network.

The shortest weighted paths identified were then compared to all KEGG pathways.

### Analysis of MyD88 and TRIF-knockout samples

An optimal gene sub-network was identified by solving the flow optimization problem using the time-course genes expression profiles from MyD88 and TRIF-knockout DCs in a manner similar to that described above for the wild-type DCs. The MyD88 gene and its interactions were removed from the starting network when the MyD88-knockout gene expression levels were considered. Similarly, during the analysis of the TRIF-knockout sample, TRIF (Ticam1) and its interactions were removed from the network.

### Enriched annotations and network representations

Enriched Gene Ontology terms and KEGG pathways were obtained using DAVID [49] with all mouse genes used as the background. Networks were prepared and formatted using Cytoscape2.7 [50]. Protein functional classes were identified using PANTHER [51].

## Supporting Information

Figure S1 Relationship between flow predicted for a gene and the average statistical significance of its occurrence in the optimal sub-network.
(PDF)

Figure S2 The fraction of identified paths of length 3 in the optimal sub-network having a certain fraction of genes from the same KEGG pathway.
(PDF)

Figure S3 Overlap between the genes within the optimal sub-network and the KEGG Toll-like receptor signaling pathway.
(PDF)

Figure S4 Overlap between the genes within the optimal sub-network and the KEGG Pathways in Cancer.
(PDF)

Figure S5 Overlap between the genes within the optimal sub-network and the KEGG Chemokine Signaling Pathway.
(PDF)

Figure S6 Overlap between the genes within the optimal sub-network and the KEGG Insulin signaling pathway.
(PDF)

Figure S7 Overlap between the genes within the optimal sub-network and the KEGG Apoptosis pathway.
(PDF)

Figure S8 Overlap between the genes within the optimal sub-network and the KEGG NF-Kappa B Signaling pathway.
(PDF)

Table S1 Genes and edges identified in the optimal sub-network for activated wild-type dendritic cells by the proposed method.
(XLSX)

Table S2 Innate immune response regulators from Amit et al. identified by the algorithm.
(XLSX)

Table S3 Regulators and TLR signature genes from Chevrier et al. identified by the network algorithm.
(XLSX)

Table S4 KEGG Pathways enriched in genes with flows greater than 1 in the predicted network.
(XLSX)

Table S5 GO Biological Process terms enriched in genes with flows greater than 1 in the predicted network.
(XLSX)

Table S6 Comparison of the enrichment and significance of the KEGG pathways predicted in all genes versus those showing differential expression.
(XLSX)

Table S7 Comparison of the enrichment and significance of the GO Biological Process terms predicted in all genes versus those showing differential expression.
(XLSX)

Table S8 Directed paths of 3 edges or more in the optimal sub-network matching directed paths of the same length in KEGG pathways.
(XLSX)

Table S9 Genes and edges identified in the optimal sub-network for activated wild-type dendritic cells by a non-temporal minimum cost flow optimization method, ResponseNet.
(XLSX)

Table S10 Genes and edges identified in the optimal sub-network for activated MyD88-knockout dendritic cells by the proposed method.
(XLSX)

Table S11 Genes and edges identified in the optimal sub-network for activated TRIF-knockout dendritic cells by the proposed method.
(XLSX)

Table S12 KEGG pathways enriched in genes identified exclusively in the network predicted for the TRIF-knockout dendritic cells.
(XLSX)

Table S13 KEGG pathways enriched in genes identified exclusively in the network predicted for the MyD88-knockout dendritic cells.
(XLSX)

Table S14 Counts of RNA-seq reads obtained before LPS stimulation and up to 8 hours after stimulation, identified and mapped.
(XLSX)

Table S15 Genes showing greater than two-fold change in expression in wild-type dendritic cells between 0.5–8 hours after LPS stimulation.
(XLSX)

Table S16 Genes showing greater than two-fold change in expression in MyD88-knockout dendritic cells between 0.5–8 hours after LPS stimulation.
(XLSX)

Table S17 Genes showing greater than two-fold change in expression in TRIF-knockout dendritic cells between 0.5–8 hours after LPS stimulation.
(XLSX)

Table S18 Number of interactions obtained from different sources used as input network to the algorithm.
(XLSX)

Table S19 Scaling of interaction scores from different source databases.
(XLSX)

Table S20 Complete network used with edge scores and weights (includes complexes as separate nodes).
(XLSX)

Table S21The values of 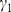 and 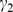 tested to obtain the optimal sub-network prediction.(XLSX)

## References

[1] Akira S, Uematsu S, Takeuchi O (2006) Pathogen recognition and innate immunity. Cell 124: 783–801. 16497588 10.1016/j.cell.2006.02.015

[2] Kawai T, Akira S (2010) The role of pattern-recognition receptors in innate immunity: update on Toll-like receptors. Nat Immunol 11: 373–384. 20404851 10.1038/ni.1863

[3] Amit I, Garber M, Chevrier N, Leite AP, Donner Y, et al (2009) Unbiased reconstruction of a mammalian transcriptional network mediating pathogen responses. Science 326: 257–263. 19729616 10.1126/science.1179050 PMC2879337

[4] Chevrier N, Mertins P, Artyomov MN, Shalek AK, Iannacone M, et al (2011) Systematic discovery of TLR signaling components delineates viral-sensing circuits. Cell 147: 853–867. 22078882 10.1016/j.cell.2011.10.022 PMC3809888

[5] Garber M, Yosef N, Goren A, Raychowdhury R, Thielke A, et al (2012) A high-throughput chromatin immunoprecipitation approach reveals principles of dynamic gene regulation in mammals. Mol Cell 47: 810–822. 22940246 10.1016/j.molcel.2012.07.030 PMC3873101

[6] Oda K, Kitano H (2006) A comprehensive map of the toll-like receptor signaling network. Mol Syst Biol 2: 2006.0015. 10.1038/msb4100057 PMC1681489 16738560

[7] Li F, Thiele I, Jamshidi N, Palsson BO (2009) Identification of potential pathway mediation targets in Toll-like receptor signaling. PLOS Comput Biol 5: e1000292. 19229310 10.1371/journal.pcbi.1000292 PMC2634968

[8] Richard G, Belta C, Julius AA, Amar S (2012) Controlling the outcome of the Toll-like receptor signaling pathways. PLOS One 7: e31341. 22363624 10.1371/journal.pone.0031341 PMC3282698

[9] Seok J, Xiao W, Moldawer LL, Davis RW, Covert MW (2009) A dynamic network of transcription in LPS-treated human subjects. BMC Syst Biol 3: 78. 19638230 10.1186/1752-0509-3-78 PMC2729748

[10] Hyduke DR, Palsson BO (2010) Towards genome-scale signalling network reconstructions. Nat Rev Genet 11: 297–307. 20177425 10.1038/nrg2750

[11] Bar-Joseph Z, Gitter A, Simon I (2012) Studying and modelling dynamic biological processes using time-series gene expression data. Nat Rev Genet 13: 552–564. 22805708 10.1038/nrg3244

[12] Liao JC, Boscolo R, Yang Y-L, Tran LM, Sabatti C, et al (2003) Network component analysis: Reconstruction of regulatory signals in biological systems. Proc Natl Acad Sci U S A 100: 15522–15527. 14673099 10.1073/pnas.2136632100 PMC307600

[13] Schulz MH, Devanny WE, Gitter A, Zhong S, Ernst J, et al (2012) DREM 2.0: Improved reconstruction of dynamic regulatory networks from time-series expression data. BMC Syst Biol 6: 104. 22897824 10.1186/1752-0509-6-104 PMC3464930

[14] Gitter A, Carmi M, Barkai N, Bar-Joseph Z (2013) Linking the signaling cascades and dynamic regulatory networks controlling stress responses. Genome Res 23: 365–376. 23064748 10.1101/gr.138628.112 PMC3561877

[15] Yeger-Lotem E, Riva L, Su LJ, Gitler AD, Cashikar AG, et al (2009) Bridging high-throughput genetic and transcriptional data reveals cellular responses to alpha-synuclein toxicity. Nat Genet 41: 316–323. 19234470 10.1038/ng.337 PMC2733244

[16] Huang SS, Fraenkel E (2009) Integrating proteomic, transcriptional, and interactome data reveals hidden components of signaling and regulatory networks. Sci Signal 2: ra40. 19638617 10.1126/scisignal.2000350 PMC2889494

[17] Huang SS, Clarke DC, Gosline SJ, Labadorf A, Chouinard CR, et al (2013) Linking proteomic and transcriptional data through the interactome and epigenome reveals a map of oncogene-induced signaling. PLOS Comput Biol 9: e1002887. 23408876 10.1371/journal.pcbi.1002887 PMC3567149

[18] Wang X, Dalkic E, Wu M, Chan C (2008) Gene module level analysis: identification to networks and dynamics. Curr Opin Biotechnol 19: 482–491. 18725293 10.1016/j.copbio.2008.07.011 PMC2615490

[19] Gu J, Chen Y, Li S, Li Y (2010) Identification of responsive gene modules by network-based gene clustering and extending: application to inflammation and angiogenesis. BMC Syst Biol 4: 47. 20406493 10.1186/1752-0509-4-47 PMC2873318

[20] Chen Y, Gu J, Li D, Li S (2012) Time-course network analysis reveals TNF-alpha can promote G1/S transition of cell cycle in vascular endothelial cells. Bioinformatics 28: 1–4. 22088844 10.1093/bioinformatics/btr619

[21] Park Y, Bader JS (2012) How networks change with time. Bioinformatics 28: i40–48. 22689777 10.1093/bioinformatics/bts211 PMC3371843

[22] Yeung KY, Dombek KM, Lo K, Mittler JE, Zhu J, et al (2011) Construction of regulatory networks using expression time-series data of a genotyped population. Proc Natl Acad Sci U S A 108: 19436–19441. 22084118 10.1073/pnas.1116442108 PMC3228453

[23] Patil A, Nakai K, Nakamura H (2011) HitPredict: a database of quality assessed protein-protein interactions in nine species. Nucleic Acids Res 39: D744–749. 20947562 10.1093/nar/gkq897 PMC3013773

[24] Yoshimura A, Naka T, Kubo M (2007) SOCS proteins, cytokine signalling and immune regulation. Nat Rev Immunol 7: 454–465. 17525754 10.1038/nri2093

[25] Karin M, Lin A (2002) NF-κB at the crossroads of life and death. Nat Immunol 3: 221–227. 11875461 10.1038/ni0302-221

[26] Zong C, Gomes AV, Drews O, Li X, Young GW, et al (2006) Regulation of Murine Cardiac 20S Proteasomes: Role of Associating Partners. Circulation Research 99: 372–380. 16857963 10.1161/01.RES.0000237389.40000.02

[27] Xie L, Liu C, Wang L, Gunawardena Harsha P, Yu Y, et al (2013) Protein Phosphatase 2A Catalytic Subunit α Plays a MyD88-Dependent, Central Role in the Gene-Specific Regulation of Endotoxin Tolerance. Cell Reports 3: 678–688. 23434512 10.1016/j.celrep.2013.01.029 PMC4060247

[28] Krüger E, Kloetzel P-M (2012) Immunoproteasomes at the interface of innate and adaptive immune responses: two faces of one enzyme. Current Opinion in Immunology 24: 77–83. 22296715 10.1016/j.coi.2012.01.005

[29] Takeda K, Akira S (2004) TLR signaling pathways. Seminars in Immunology 16: 3–9. 14751757 10.1016/j.smim.2003.10.003

[30] Cai S, Batra S, Shen L, Wakamatsu N, Jeyaseelan S (2009) Both TRIF- and MyD88-Dependent Signaling Contribute to Host Defense against Pulmonary Klebsiella Infection. The Journal of Immunology 183: 6629–6638. 19846873 10.4049/jimmunol.0901033 PMC2777750

[31] Narasimamurthy R, Hatori M, Nayak SK, Liu F, Panda S, et al (2012) Circadian clock protein cryptochrome regulates the expression of proinflammatory cytokines. Proc Natl Acad Sci U S A 109: 12662–12667. 22778400 10.1073/pnas.1209965109 PMC3411996

[32] Kawai T, Akira S (2011) Regulation of innate immune signalling pathways by the tripartite motif (TRIM) family proteins. EMBO Molecular Medicine 3: 513–527. 21826793 10.1002/emmm.201100160 PMC3377094

[33] Patil A, Nakamura H (2005) Filtering high-throughput protein-protein interaction data using a combination of genomic features. BMC Bioinformatics 6: 100. 15833142 10.1186/1471-2105-6-100 PMC1127019

[34] Zheng Y, Zhang C, Croucher DR, Soliman MA, St-Denis N, et al (2013) Temporal regulation of EGF signalling networks by the scaffold protein Shc1. Nature 499: 166–171. 23846654 10.1038/nature12308 PMC4931914

[35] Yamashita R, Sathira NP, Kanai A, Tanimoto K, Arauchi T, et al (2011) Genome-wide characterization of transcriptional start sites in humans by integrative transcriptome analysis. Genome Res 21: 775–789. 21372179 10.1101/gr.110254.110 PMC3083095

[36] Adachi O, Kawai T, Takeda K, Matsumoto M, Tsutsui H, et al (1998) Targeted Disruption of the MyD88 Gene Results in Loss of IL-1- and IL-18-Mediated Function. Immunity 9: 143–150. 9697844 10.1016/s1074-7613(00)80596-8

[37] Yamamoto M, Sato S, Hemmi H, Hoshino K, Kaisho T, et al (2003) Role of Adaptor TRIF in the MyD88-Independent Toll-Like Receptor Signaling Pathway. Science 301: 640–643. 12855817 10.1126/science.1087262

[38] Coordinators NR (2013) Database resources of the National Center for Biotechnology Information. Nucleic Acids Res 41: D8–D20. 23193264 10.1093/nar/gks1189 PMC3531099

[39] Langmead B, Trapnell C, Pop M, Salzberg SL (2009) Ultrafast and memory-efficient alignment of short DNA sequences to the human genome. Genome Biol 10: R25. 19261174 10.1186/gb-2009-10-3-r25 PMC2690996

[40] Trapnell C, Pachter L, Salzberg SL (2009) TopHat: discovering splice junctions with RNA-Seq. Bioinformatics 25: 1105–1111. 19289445 10.1093/bioinformatics/btp120 PMC2672628

[41] Roberts A, Pimentel H, Trapnell C, Pachter L (2011) Identification of novel transcripts in annotated genomes using RNA-Seq. Bioinformatics 27: 2325–2329. 21697122 10.1093/bioinformatics/btr355

[42] Breuer K, Foroushani AK, Laird MR, Chen C, Sribnaia A, et al (2013) InnateDB: systems biology of innate immunity and beyond—recent updates and continuing curation. Nucleic Acids Res 41: D1228–D1233. 23180781 10.1093/nar/gks1147 PMC3531080

[43] Matys V, Kel-Margoulis OV, Fricke E, Liebich I, Land S, et al (2006) TRANSFAC® and its module TRANSCompel®: transcriptional gene regulation in eukaryotes. Nucleic Acids Res 34: D108–D110. 16381825 10.1093/nar/gkj143 PMC1347505

[44] Kanehisa M, Goto S (2000) KEGG: Kyoto Encyclopedia of Genes and Genomes. Nucleic Acids Res 28: 27–30. 10592173 10.1093/nar/28.1.27 PMC102409

[45] Kanehisa M, Goto S, Sato Y, Furumichi M, Tanabe M (2012) KEGG for integration and interpretation of large-scale molecular data sets. Nucleic Acids Res 40: D109–D114. 22080510 10.1093/nar/gkr988 PMC3245020

[46] Mahadevan R, Schilling CH (2003) The effects of alternate optimal solutions in constraint-based genome-scale metabolic models. Metab Eng 5: 264–276. 14642354 10.1016/j.ymben.2003.09.002

[47] Dijkstra EW (1959) A note on two problems in connexion with graphs. Numerische Mathematik 1: 269–271.

[48] Opsahl T, Agneessens F, Skvoretz J (2010) Node centrality in weighted networks: Generalizing degree and shortest paths. Social Networks 32: 245–251.

[49] Huang DW, Sherman BT, Lempicki RA (2008) Systematic and integrative analysis of large gene lists using DAVID bioinformatics resources. Nat Protocols 4: 44–57. 10.1038/nprot.2008.211 19131956

[50] Saito R, Smoot ME, Ono K, Ruscheinski J, Wang P-L, et al (2012) A travel guide to Cytoscape plugins. Nat Meth 9: 1069–1076. 10.1038/nmeth.2212 PMC3649846 23132118

[51] Mi H, Muruganujan A, Thomas PD (2013) PANTHER in 2013: modeling the evolution of gene function, and other gene attributes, in the context of phylogenetic trees. Nucleic Acids Res 41: D377–D386. 23193289 10.1093/nar/gks1118 PMC3531194

