# Supplementary material for: Linking Transcriptional Changes over Time in Stimulated Dendritic Cells to Identify Gene Networks Activated during the Innate Immune Response
Source: PLoS Comput Biol. 2013 Nov 7;9(11):e1003323. doi: 10.1371/journal.pcbi.1003323 (PMC3820512; doi:10.1371/journal.pcbi.1003323)

Supporting Figure 1. Relationship between flow predicted for a gene and the average statistical significance of its occurrence in the optimal subnetwork

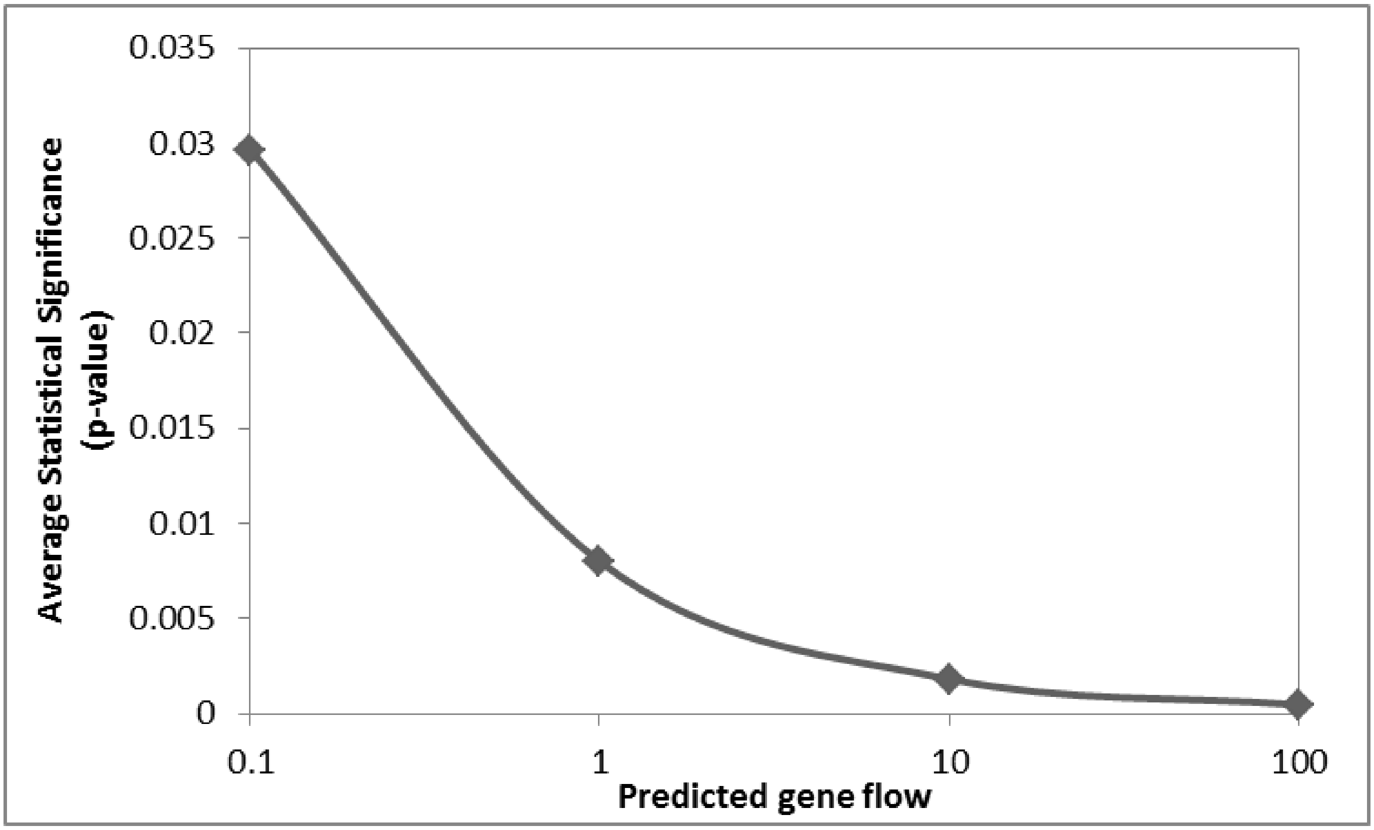

Supplement: Figure S1 — Relationship between flow predicted for a gene and the average statistical significance of its occurrence in the optimal sub-network. (PDF) [file pcbi.1003323.s001.pdf]
