# Supplementary material for: Linking Transcriptional Changes over Time in Stimulated Dendritic Cells to Identify Gene Networks Activated during the Innate Immune Response
Source: PLoS Comput Biol. 2013 Nov 7;9(11):e1003323. doi: 10.1371/journal.pcbi.1003323 (PMC3820512; doi:10.1371/journal.pcbi.1003323)

Supporting Figure S2. The fraction of identified paths of length 3 in the optimal subnetwork having a certain fraction of genes from the same KEGG pathway.

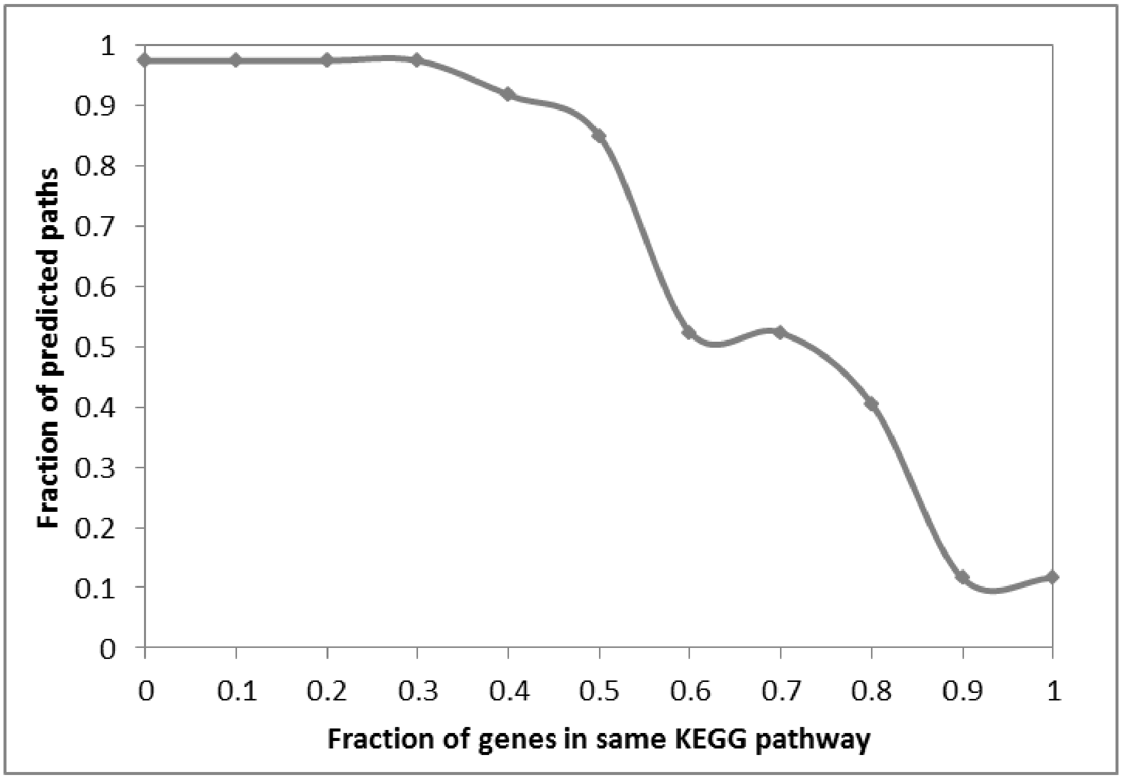

Supplement: Figure S2 — The fraction of identified paths of length 3 in the optimal sub-network having a certain fraction of genes from the same KEGG pathway. (PDF) [file pcbi.1003323.s002.pdf]
