# Supplementary material for: Linking Transcriptional Changes over Time in Stimulated Dendritic Cells to Identify Gene Networks Activated during the Innate Immune Response
Source: PLoS Comput Biol. 2013 Nov 7;9(11):e1003323. doi: 10.1371/journal.pcbi.1003323 (PMC3820512; doi:10.1371/journal.pcbi.1003323)

Supporting Figure S5. Overlap between the genes within the optimal subnetwork and the KEGG Chemokine Signaling Pathway. (Pink: 0.5-1hr, Yellow: 2-4 hrs, Mintgreen: 6-8hrs, Blue: identified, Green: Not in optimal sub-network)

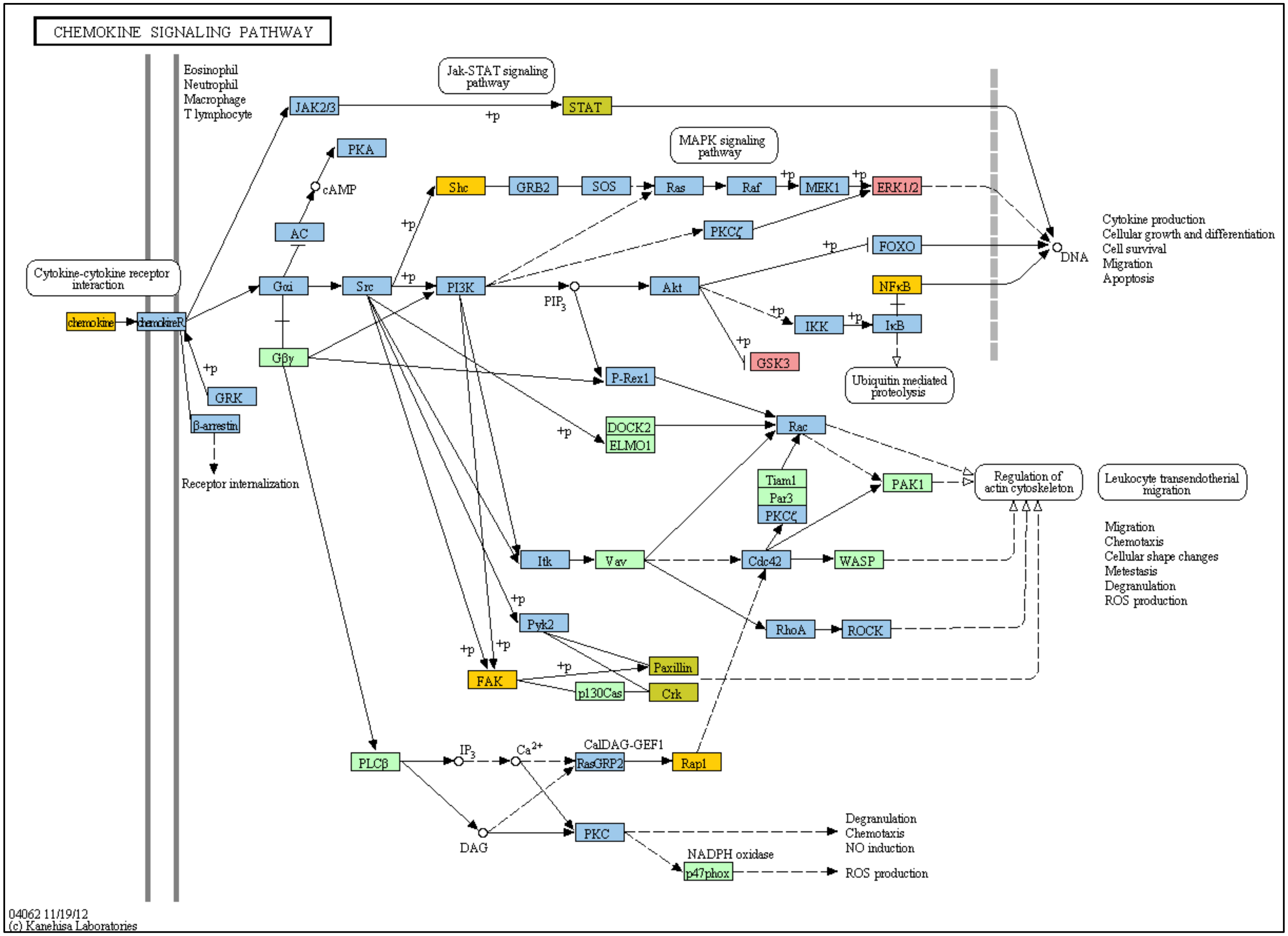

Supplement: Figure S5 — Overlap between the genes within the optimal sub-network and the KEGG Chemokine Signaling Pathway. (PDF) [file pcbi.1003323.s005.pdf]
